# Antipsychotics for the Treatment of Behavioral and Psychological Symptoms of Dementia (BPSD)

**DOI:** 10.2174/157015908784533860

**Published:** 2008-06

**Authors:** Rosa Liperoti, Claudio Pedone, Andrea Corsonello

**Affiliations:** 1 Centro di Medicina dell'Invecchiamento, Dipartimento di Scienze Gerontologiche, Geriatriche e Fisiatriche, Università Cattolica del Sacro Cuore, Largo A. Gemelli, I-00168 Rome, Italy; 2 Area di Geriatria, Università Campus BioMedico, Via dei Compositori, I-00100 Rome, Italy; 3 Fondazione San Raffaele, Cittadella della Carità, I-74100 Taranto, Italy; 4 Istituto Nazionale di Ricovero e Cura per Anziani (INRCA), I-87100 Cosenza, Italy

**Keywords:** Behavioral and psychological symptoms of dementia (BPSD), dementia, antipsychotics.

## Abstract

Behavioral and psychological symptoms of dementia (BPSD), i.e. verbal and physical aggression, agitation, psychotic symptoms (hallucinations and delusions), sleep disturbances, oppositional behavior, and wandering, are a common and potentially severe problem complicating dementia. Their prevalence is very high and it is estimated that up to 90% of patients with Alzheimer’s disease (AD) may present at least one BPSD. Beside the obvious impact on the quality of life of people with dementia, BPSD are responsible for increased risk of patient institutionalization and increased costs. Furthermore, they are associated with caregivers’ stress and depression. Drugs used include antipsychotics, antidepressants, anticonvulsivants, anxiolytics, cholinesterase inhibitors and N-methyl-D-aspartate receptor modulators. Among these, the most commonly used are anti-psychotics. These drugs have been used for many decades, but in the last years new compounds have been marketed with the promise of comparable efficacy but less frequent adverse effects (especially extra-pyramidal side effects). Their safety, however, has been challenged by data showing a potential increase in adverse cerebrovascular side effects and mortality. This review will summarize the pathophysiology and neuropharmacology of BPSD, it will describe the characteristics of the anti-psychotics most commonly used focusing on their efficacy and safety in BPSD.

## INTRODUCTION

Dementia is one of the most important problems in clinical practice given the growth of elderly populations in the last decades [33]. While the hallmark of all dementia syndromes is the decline of varying cognitive abilities, cognitive impairment should not be considered the only important symptomatology present in dementia. The term "behavioural and psychological symptoms of dementia" (BPSD) describes a wide spectrum of non-cognitive manifestations of dementia, including verbal and physical aggression, agitation, psychotic symptoms (hallucinations and delusions), sleep disturbances, oppositional behavior, and wandering [7, 18]. 

BPSD are very frequent in dementia: up to 90% of patients with Alzheimer’s disease (AD) may present at least one of these symptoms, and it can be estimated that about one-third of AD patients have severe problems [10, 16, 45, 61, 62]. Agitation, aggressiveness, wandering, oppositional behaviour and psychotic disorders are present in about 10-50% of patients with AD, with a considerable impact on the functional status [8, 9, 14, 25, 26, 45, 54]. Indeed, these symptoms are very invasive and difficult to manage by the caregivers and the care teams [14, 16, 61]. 

BPSD may not be obvious during the early stages of AD, but these symptoms are also present in dementia syndromes other than AD, such as dementia with Lewy bodies or frontotemporal lobar degeneration [63, 68], where they may appear also in the early stages. Thus, the course of BPSD differ according to the type of dementia [11, 20, 55]. Finally, the appearance of psychotic symptoms is a relevant risk factor for the development of aggressive behaviour and agitation, and is associated with a poorer functional prognosis [20, 54].

Anyway, the appearance of BPSD during the evolution of any dementia syndrome represents a major management problem for both physicians and caregivers, and the evolution of these symptoms is the major reason for caregiver “burnout” and patient institutionalization, with an enormous increase in medical and indirect costs, and with a frequent decrease in quality of life for the patient and the caregiver [7, 17, 33].

The etiology of these symptoms is yet to be definitely clarified, and this certainly contributes to limit the pharmacological approach to BPSD. Nonetheless, it is conceivable that several factors including neurobiological, physical and environmental components are likely to be involved [7]. We will focus on the pathophysiological mechanisms underlying BPSD and the neuropharmacology of drugs used for the treatment of these symptoms, and we will also discuss the available evidence regarding the efficacy and the safety of conventional and atypical antipsychotics in patients with BPSD.

## PATHOPHYSIOLOGY OF BPSD

There is convincing evidence that the origin of BPSD in AD derives from identifiable anatomical and biochemical abnormalities. Given the wide array of psychopathologic symptoms in AD, however, it is unlikely that lesions of specific brain structure is related with a specific BPSD. Furthermore, it is likely that baseline psychological factors are involved along with biological factors in the appearance of behavioural problems [65], although a retrospective bias can be in part responsible for the observed associations [79].

Genetic studies show that chromosomal abnormalities are a risk factor for the development of BPSD. For example, a relationship between presenilin 1 and psychosis has been demostrated [39]. An association has also been shown between polymorphism of serotonin receptors genes (5HT_2A_ 102-T/C and 5HT_2C_ Cys23Ser) and visual and auditory hallucinations, with the two polymorphisms having an additive effect on visual hallucinations [44]. Polymorphism of the dopamine receptors genes is also involved: in 275 outpatients with probable AD, homozygous for DRD1 allele B1 and homozygous for either DRD3 allele were both associated with psychosis [83].

A genetic polymorphism of the serotonin transporter promoter region (L/L genotype) has been implicated with aggressive behaviour in patients with AD [81]. The same genotype seems to be associate with a distinctive phenotype characterized by psychotic symptoms and aggressive behaviour [84].

The typical pathologic lesions of AD, neurofibrillary tangles (NFT), exhibit a characteristic distribution pattern that is correlated with dementia stage. In the earlier stages, there is an invasion from the enthorinal cortex to the hippocampus, while in more advanced stages there also is an involvement of the neo-cortex [12]. While “negative” psychiatric symptoms (such as depression) can be evident even before a diagnosis of AD is made, “positive” symptoms (agitation, aggressive behaviour) appear usually at later stages of dementia, after the appearance of cognitive abnormalities and presumably when the neo-cortex is invaded by NFT [47]. 

As can be expected, different behavioural problems reflect involvement of different cerebral areas. It has been shown that people with AD who develop psychosis have a 2.3-fold greater density of NFT in the neo-cortex (middle frontal, anterior third of the superior temporal, inferior parietal) compared to AD patients who will not develop psychosis [29]. Neurofunctional imaging studies have shown that psychosis in probable AD is associated with a reduction in prefrontal, left frontal-temporal, and right parietal metabolism [61,82]. These evidences, however, come from studies with very small sample size.

Delusional misidentification (such as the belief that a close relative has been replaced by some other person having the same appearance) have been found to be correlated with lower neurone count in the CA1 area of the hippocampus; in the same study a lower neurone count in the dorsal raphe was associated with delusions and hallucinations [34].

Higher NFT concentration has been reported in the orbito-frontal cortex of AD patients with agitation [85], while single-photon emission CT demonstrated hypoperfusion of the left anterior temporal cortex in AD patients showing aggressive behaviour [43]. Also in non-AD dementia, there seems to be an association between location of pathologic lesions and behavioural problems. In Lewy’s disease, for instance, it has been shown that there is a strong association between the density of Lewy’s bodies in the amygdala and parahyppocampal cortex and the presence of severe visual hallucination [38]. 

## PHARMACOLOGY OF ANTIPSYCHOTICS

As different cerebral regions are involved in the pathogenesis of BPSD, different neurotransmitters have been found to be implicated in these disturbances (acetylcholine, dopamine, serotonine). Most pharmacologic treatment for BPSD are based else on drugs increasing the activity of these neurotransmitters (acetylcholine), or by decreasing or modulating it (dopamine, serotonine).

For decades, the mainstay of treatment for psychosis in dementia have been the so-called “conventional” or first-generation antipsychotics, that have been used since the ‘50s. There are three principal chemical classes of these drugs (phenotiazines, butyrophenones and thioxanthenes), and all of them share high affinity for the D2 dopamine receptor. The efficacy of these drugs is strictly correlated with the occupancy rate of D2 receptors: a PET study demonstrated that D2 occupancy predicted the clinical response to haloperidol, and that a threshold of 65% occupancy rate provided a good separation between responders and non-responders [50]. These results are consistent with others obtained with a different antipsychotic drug (raclopride) [70]. The occupancy of D2 receptors in the basal ganglia is also correlated with extra-pyramidal effects of these drugs [30, 70], as well as to other side effects such as hyperprolactinemia [50]. First-generation anti-psychotics have been also shown to block D2 receptors in the limbic cortical areas, and this activity seems to be most important in treating psychotic symptoms [89].

Newer anti-psychotic drugs have been dubbed “atypical” because they seem to induce extra-pyramidal effects less frequently and because they seem to have therapeutic efficacy in patients who do not respond to first-generation antipsychotics.

Clozapine is the prototype of this newer anti-psychotic agents. Its pharmacodynamic profile includes low affinity for both D1 and D2 dopamine receptors, along with high affinity for D4 dopamine receptor and serotonine receptors (5HT2 and 3) [51]. It also has an anti-glutamatergic action [56], as well as alpha-2 receptor affinity and M1 cholinergic receptor blocking activity [28]. Dopamine receptors block by clozapine is evident especially in the mesolimbic, but not in the nigro-striatal system [3], and this can in part account for the low incidence of extrapyramidal side effects.

Enhanced efficacy and reduced extra-pyramidal symptoms observed with second-generation anti-psychotic drugs have been explained with their high 5HT2/D2 activity [67]. More recently, the fast dissociation of “atypical” anti-psychotics from the D2 receptor compared to older drugs has been proposed as the mechanism by which these drug have an anti-psychotic effect without extra-pyramidal effects or prolactine elevation [49]. Despite its seemingly optimal pharmacological profile, use of clozapine is not widespread because a consistent risk of agranulocytosis. 

Risperidone has been marketed as an atypical antipsychotic because of its 5HT2/D2 affinity ratio. However, it has been shown a similar proportion of D2 receptors occupied by risperidone and haloperidol [48]. Olanzapine was introduced shortly after risperidone, and has a pharmacologic profile similar to clozapine [28]. Quetiapine was the fourth “atypical” anti-psychotic marketed, it has a chemical structure and multi-receptor activity similar to clozapine [72], and acts selectively in the limbic system [46]. Ziprasidone, although structurally different from other antipsychotics, also shows a high 5HT/D2 ratio, although its affinity for D2 receptors is high [32].

Benzamides (sulpiride and amisulpiride) are highly selective antagonistis of dopamine at the D2 and D3 receptors with a preferential limbic activity. Besides this post-synaptic actions, it has been shown to antagonize the pre-synaptic receptors that modulate dopamine release [77].

Aripiprazole is a newer antipsychotic agent with a mechanism of action that is different from both first and second generation antipsychotics. It acts as a partial agonist at the D2 and 5HT1A receptors and as an antagonist at the 5HT2A receptor, and is considered a “stabilizer” of the dopamine/serotonin system [15].

## EFFICACY OF ANTIPSYCHOTICS

Conventional antipsychotics have been approved in the 1950s mainly for the treatment of schizophrenia. Since then, these agents have been systematically used for the treatment of BPSD in spite of a substantial lack of scientific evidence supporting their use in dementia. Few trials investigating the efficacy of conventional agents for the treatment of BPSD have been conducted between the 1960s and the late 1980s [6, 60, 75]. These studies mainly focused on the effect of haloperidol and thioridazine. They were characterized by small sample sizes and possible lack of power. Data from these early studies showed a modest advantage of conventional antipsychotics over placebo with a nearly 40% placebo response, and only 18% benefit over placebo in the meta-analysis by Schneider *et al.* [75]. Also, according to some of these studies, the observed superiority of conventional antipsychotics over placebo would be limited to symptoms of aggression and it would be absent in other behavioural and psychotic symptoms [60].

Atypical antipsychotics have been licensed in the 1990s and approved by The US Food and Drug Administration (FDA) exclusively for the treatment of schizophrenia. Rapidly after their introduction in clinical practice, these medications have become the new standard of care for BPSD due to their reported advantages over conventional agents, particularly with respect to extrapyramidal symptoms (EPS) and tardive diskinesia [19, 35, 68]. Over the last decade, the off-label use of atypical antipsychotics in dementia has been promoted by clinical practice guidelines although the limited number of clinical trials suggesting the efficacy of these agents in dementia [2, 37]. In the late 1990s, atypical agents accounted for more than 80% of antipsychotic prescriptions in dementia [37, 59]. To date 22****randomized placebo-controlled trials have investigated the efficacy of atypical antipsychotics for the treatment of BPSD. Only eleven****of these studies have been published in full at the time of completion of this review (Table **1**). No data from double blind randomized****clinical trials on patients with dementia are available for amisulpride, clozapine, sertindole, ziprasidone or zotepine.

Compared to placebo, risperidone has been shown to be beneficial on psychotic symptoms and aggression at doses of 1 mg and 2 mg per day in three placebo controlled clinical trials [13, 23, 52]. These studies were conducted on patients with Alzheimer’s disease, vascular dementia or mixed dementia on a 12 weeks time-period. 

Olanzapine has been shown significantly effective for improving behavioural symptoms, psychosis and aggression at 5 to 10 mg per day dose compared with placebo [22, 80]. This evidence derives from two randomized placebo-controlled clinical trials conducted among patients with dementia for a 10 week and 6 week period of time respectively [22, 80]. In contrast with these data, a recent study on patients with moderate to severe psychotic symptoms of dementia randomly assigned to receive a flexible dose of olanzapine (2.5-10.g per day), risperidone (0.5-2 mg per day) or placebo demonstrated similar improvement of BPSD in the three treatment groups with higher discontinuation rate due to adverse events in the olanzapine and risperidone groups [24]. 

Very recently, a 10-week, double- blind, placebo-controlled study has shown that quetiapine at a fixed dose of 200 mg per day is effective and well tolerated for treating agitation in institutionalized patients with dementia [90]. Also, one published study involving a small number of patients with dementia has demonstrated no effect of quetiapine or rivastigmine on improving clinically significant agitation and an increased cognitive decline associated with the use of quetiapine [4]. Finally, a small double-blind, placebo-controlled, randomized study has shown no significant difference between quetiapine and placebo in terms of efficacy on controlling agitation and psychosis among patients with dementia and parkinsonism [53]. In this study quetiapine was well tolerated and did not worse parkinsonism. As pointed out by the authors of this study, lack of power and a large placebo effect may have contributed to the resulted lack of efficacy. 

A single double blind, randomized, placebo-controlled clinical trial has investigated the efficacy and safety of aripiprazole in patients with Alzheimer’s disease and psychosis [21]. According to this 10-week study, aripiprazole at a mean dose of 10 mg per day appeared to confer no benefit over placebo for controlling delusions and hallucinations and it was well tolerated. 

Very recently, the results of a large effectiveness trial, the CATIE-AD (Clinical Antipsychotic Trials of Intervention Effectiveness-Alzheimer’s Disease), have been published. According to this multicenter, double-blind, placebo-con-trolled trial on outpatients with Alzheimer’s disease and psychosis, aggression or agitation, the effect of olanzapine (mean dose 5.5 mg per day) and risperidone (mean dose 1.0 mg per day) in treating neuropsychiatric symptoms was equally beneficial and superior to the effect of placebo and quetiapine (mean dose 56.5 mg per day) [76]. However, these benefits were evident only among those patients who tolerated these medications and did not discontinue them due to side effects. Similar rates of treatment discontinuation were reported in the different study groups. However, patients on antipsychotics discontinued mostly because of adverse effects while patients on placebo discontinued mostly because of lack of efficacy. According to authors’ conclusions potential side effect associated with antipsychotic medications in dementia may outweigh possible benefits. 

A comprehensive review of the available randomized, double blind, placebo-controlled trials has been conducted by Ballard and White for the Cochrane collaboration to determine the effectiveness of atypical antipsychotics for the treatment of psychiatric and behavioural symptoms in Alzheimer’s disease [5]. The authors analyzed sixteen placebo-controlled trials among which only six studies were published in full in peer reviewed journals. According to the Cochrane authors, evidence suggests that both risperidone and olanzapine may reduce aggression and risperidone may also reduce psychosis compared to placebo. However, an increased risk of extrapyramidal symptoms and adverse cerebrovascular events associated with atypical antipsychotics would outweigh the modest effectiveness of these medications. According to Cochrane findings, due to the increased risk of adverse effects, the use of atypical antipsychotics in clinical practice would not be suitable and should be limited to those patients presenting with significant distress and risk associated with symptoms. 

## SAFETY OF ANTIPSYCHOTICS

At the time of their introduction in clinical practice, atypical antipsychotics have been reported to be characterized by a better safety profile compared to conventional medications, especially with respect to extrapyramidal symptoms such as parkinsonism and tardive diskinesia. Data from clinical trials substantially confirm the superior EPS profile of atypical over conventional antipsychotics [13, 22, 23, 52, 80]. Risperidone at dose of 1 mg per day has been proven to cause less EPS compared with placebo and haloperidol [13, 23, 52]. However, this relative benefit of risperidone relative to haloperidol disappeared at dosages of 2 mg per day or higher [52]. In two randomized trials of olanzapine there was no increased incidence of EPS in the olanzapine groups (at doses of 5 to 15 mg per day) compared with the placebo group. Observational data supported findings from clinical trials [22, 80]. Overall, the available evidence suggests that in dementia patients EPS are less frequently associated with atypical antipsychotics relative to conventional agents. However, parkinsonism and tardive diskinesia may be caused by atypical antipsychotics, especially at high dosages.

Beginning in 2002, warnings about a possible increased risk of cerebrovascular events (CVEs) and death among patients with dementia being treated with risperidone or olanzapine have been issued by drugs’ manufacturers and health regulatory agencies worldwide [31, 41, 87, 88]. These concerns arose from the revision of both published and unpublished clinical trials on atypical antipsychotics among patients with dementia. According to the results of these analyses, risperidone was associated with a nearly 3-fold increase in the risk of CVEs in dementia patients [41]. The risk associated with risperidone did not differ from the risk associated with placebo when serious CVEs including death, life threatening or leading to permanent disability events were considered. Also, a nearly 2-fold increase in the risk of CVEs associated with olanzapine was calculated but this estimate did not reach statistical significance [41]. More recently, a meta-analysis of 15 randomized clinical trials on patients with dementia being treated with atypical antipsychotics reported a 65% increased all cause mortality associated with atypical antipsychotics compared with placebo [74]. In spite of this growing evidence from clinical trials, data from several large observational studies failed to support the conclusion of a possible increased risk of CVEs and death associated with atypical antipsychotics [36, 42, 57]. Moreover, a recent large retrospective cohort study suggested that conventional antipsychotics may indeed carry a nearly 40% excess risk of death compared with atypical agents and therefore they would not represent a valid alternative to atypical compounds for the treatment of BPSD [86]. According to the available evidence, in April 2005, the FDA has issued a public health advisory to warn about a possible increased risk of death associated with atypical antipsychotics [31]. The FDA has asked manufacturers of these drugs to include a boxed warning in their labeling describing the risk and has reminded that atypical antipsychotics have not been approved for dementia which represents an off-label indication. The FDA has also anticipated a possible future extension of this warning to conventional antipsychotics. 

Treatment with atypical antipsychotics among patients with dementia has been linked to some risk of lengthening of QTc interval at EKG [40, 78]. However, data available have suggested that atypical antipsychotics may not increase the risk of clinical outcomes related to QTc prolongation, including ventricular arrhythmias and sudden death and with respect to cardiac toxicity they may be safer than conventional medications [57, 71].

Atypical antipsychotics are known to cause a spectrum of metabolic adverse effects such as diabetes, hyperlipidaemia and weight gain among young and adult patients with schizophrenia [66]. In particular, the risk of weight gain, diabetes and hyperlipidaemia has been reported high for clozapine and olanzapine, moderately high for quetiapine and low for risperidone.****To date, there is little evidence that patients with dementia being treated with atypical antipsychotics may experience such metabolic effects [13, 22, 23, 52, 80]. Also no data are available to investigate the extent to which such metabolic disturbances may contribute to the possible cardiovascular toxicity associated with atypical antipsychotics.

## CONCLUSIONS

BPSD represent one of the main mental issues in the geriatric population. To date, limited evidence supports available therapeutic strategies and current recommendations mainly derive from consensus of experts [1, 7, 27]. Based on safety considerations and in light of the high rates of placebo-response in clinical trials, non pharmacological approaches are generally recognized as the first-line strategy for the treatment of BPSD. The pharmacological approach based on the use of antipsychotic medications is recommended for the short-term treatment (up to three months) and among those patients who manifest severe symptoms that may cause extreme distress and harm to patients or others. Antipsychotic prescription is limited and strictly regulated in many countries and BPSD are still an off-label indication for most of these agents.****

In clinical practice, the therapeutic choice between atypical and conventional medications should be based on a careful evaluation of the potential benefits and risks of both classes of antipsychotics as well as patients’ individual risk profile. Physicians should evaluate each patient individually and judge whether or not the magnitude of the potential risks outweighs the benefits that can be expected from the use of antipsychotics. 

Finally, evidence for potential pharmacological alternatives to antipsychotics is also lacking. In the near future, research efforts should be directed to investigate and identify alternative therapeutic strategies which may combine non pharmacological treatments with drug therapy and need to be tailored to patients with dementia and their families.

## Figures and Tables

**Table 1.** Published Randomized Clinical Trials of Atypical Antipsychotics among Patients with BPSD

Trial	Intervention	Daily Dose	Population	Setting	Duration (Weeks)	Primary Efficacy Measure
Katz,1999 [52]	risperidone *vs* placebo	Fixed,0.5,1.0 or 2.0 mg	N=625;AD,VaD, mixed	Nursing home	12	BEHAVE-AD
De Deyn 1999 [23]	risperidone *vs* haloperidol*vs* placebo	Flexible, mean risperidone 1.1 mg, mean haloperidol 1.2 mg	N=344;AD, VaD, mixed	Nursing home	13	BEHAVE-AD,CMAI, CGI
Brodaty, 2003 [13]	risperidone *vs* placebo	Flexible, mean 0.95 mg	N=309;AD, VaD, mixed	Nursing home	12	CMAI, BEHAVE-AD, CGI
Street, 2000 [80]	olanzapine *vs* placebo	Fixed, 5.0, 10.0 or 15.0 mg	N=206; AD	Nursing home	6	NPI-NH
De Deyn,2004 [22]	olanzapine *vs* placebo	Fixed,1.0, 2.5, 5.0 or 7.5mg	N=652;AD	Nursing home	10	NPI-NH
Deberdt,2005 [24]	olanzapine *vs* placebo *vs*risperidone	Flexible, mean olanzapine 5.2 mg, mean risperidone 1.0 mg	N=298; AD, VaD,mixed	Outpatient and residential	10	NPI, CGI
Ballard,2005 [4]	quetiapine *vs* rivastigmine *vs*placebo	Flexible,range quetiapine 50-100 mg	N=93;AD	Nursing home	26	CMAI,SIB
De Deyn, 2005 [21]	aripiprazole *vs* placebo	Fixed, 5, 10 or 15 mg	N=208; AD	Outpatient	10	NPI, BPRS
Schneider, 2006 [76]	olanzapine *vs* quetiapine*vs* risperidone *vs* placebo	Flexible, mean olanzapine 5.5 mg, mean quetiapine 56.5 mg, mean risperidone 1.0 mg)	N=421; AD	Outpatient	36	Time from initial treatment to discontinuation/CGIC
Zhong, 2007 [90]	quetiapine *vs*. placebo	Fixed, 100 or 200 mg	N=333; AD	Nursing home	10	PANSS-EC, CGI,NPI-NH, CMAI
Kurlan, 2007[53]	quetiapine *vs*. placebo	Flexible, mean 120 mg	N=40; DLB, PD, AD	Outpatient	10	BPRS

**Abbreviations:** AD= Alzheimer’s disease; VaD=Vascular dementia; DLB=Dementia with Lewy Bodies; PD= Parkinson disease with dementia; BEHAVE-AD=Behavioral Pathology in Alzheimer’s disease rating scale; CMAI= Cohen-Mansfield Agitation Inventory; CGI=Clinical Global Impression; NPI= Neuropsychiatric Inventory; NPI-NH=Neuropsychiatric Inventory –nursing home version; SIB= severe impairment battery; BPRS= Brief Psychiatric Rating Scale; CGIC=Clinical Global Impression of Change; PANSS-EC= positive and negative syndrome scale-excitement component.
